# Involvement of 5-HT_1A_ Receptors in the Anxiolytic-Like Effects of Quercitrin and Evidence of the Involvement of the Monoaminergic System

**DOI:** 10.1155/2016/6530364

**Published:** 2016-05-19

**Authors:** Jian Li, Qian-tong Liu, Yi Chen, Jie Liu, Jin-li Shi, Yong Liu, Jian-you Guo

**Affiliations:** ^1^School of Chinese Materia Medica, Beijing University of Chinese Medicine, Beijing 100102, China; ^2^Key Laboratory of Mental Health, Institute of Psychology, Chinese Academy of Sciences, Beijing 100101, China

## Abstract

Quercitrin is a well-known flavonoid that is contained in Flos Albiziae, which has been used for the treatment of anxiety. The present study investigated the anxiolytic-like effects of quercitrin in experimental models of anxiety. Compared with the control group, repeated treatment with quercitrin (5.0 and 10.0 mg/kg/day, p.o.) for seven days significantly increased the percentage of entries into and time spent on the open arms of the elevated plus maze. In the light/dark box test, quercitrin exerted an anxiolytic-like effect at 5 and 10 mg/kg. In the marble-burying test, quercitrin (5.0 and 10.0 mg/kg) also exerted an anxiolytic-like effect. Furthermore, quercitrin did not affect spontaneous locomotor activity. The anxiolytic-like effects of quercitrin in the elevated plus maze and light/dark box test were blocked by the serotonin-1A (5-hydroxytryptamine-1A (5-HT_1A_)) receptor antagonist WAY-100635 (3.0 mg/kg, i.p.) but not by the *γ*-aminobutyric acid-A (GABA_A_) receptor antagonist flumazenil (0.5 mg/kg, i.p.). The levels of brain monoamines (5-HT and dopamine) and their metabolites (5-hydroxy-3-indoleacetic acid, 3,4-dihydroxyphenylacetic acid, and homovanillic acid) were decreased after quercitrin treatment. These data suggest that the anxiolytic-like effects of quercitrin might be mediated by 5-HT_1A_ receptors but not by benzodiazepine site of GABA_A_ receptors. The results of the neurochemical studies suggest that these effects are mediated by modulation of the levels of monoamine neurotransmitters.

## 1. Introduction

Anxiety is a state of excessive fear and characterized by psychomotor tension, sympathetic hyperactivity, and vigilance [1]. More than one of every four adults experience at least one anxiety disorder in his or her lifetime [2]. Benzodiazepines are the most common drugs that have been used for the treatment of anxiety, but these compounds have obvious side effects, such as sedation, muscle relaxation, amnesia, and dependence potential [3–5]. Therefore, more efficacious and better-tolerated anxiolytic agents need to be developed.

*Albizia julibrissin* Durazz., commonly called mimosa or silk trees, is widely distributed across China, Africa, Mid-Asia, East Asia, and North America [6]. Flos Albiziae is the dry flowers or flower buds of* Albizia julibrissin *Durazz., which have been used for the treatment of insomnia, amnesia, sore throat, and contusion in traditional oriental medicine [7]. Our previous study showed that the total flavones contained in* Albizia julibrissin* exerted anxiolytic effects [8]. Quercitrin is the major component of flavonoids contained in Flos Albiziae [9]. Quercitrin has been reported to have many biological properties, including sedative [10], neuroprotective [11], anti-inflammatory [12, 13], antinociceptive [14], and antileishmanial [15] effects. However, the anxiolytic potential of quercitrin has not yet been reported.

Many currently available anxiolytic drugs are known to act through GABA-ergic or serotonergic systems [16, 17]. GABA plays an important role in anxiety disorders. Studies that use knockout and knock-in mice have been conducted to clarify the role of the GABA_A_ receptor complex in the pathophysiology and management of anxiety [18]. Serotonin (5-hydroxytryptamine (5-HT)) is a key modulatory neurotransmitter that has been implicated in the pathophysiology and treatment of anxiety and mood disorders [19]. The proposed role of 5-HT_1A_ receptors in modulating anxiety-related behavior is supported by recent studies that employed 5-HT_1A_ receptor knockout mice [20, 21]. In addition, clinical and animal studies have provided evidence to support the involvement of central neurochemical systems, including neurotransmitter, neuromodulator, and neuroendocrine systems, in anxiety disorders [22]. Modulation of the monoaminergic system forms the basis for the actions of anxiolytic drugs [22], providing a framework for studying the pathophysiology of anxiety disorders and pharmacotherapies that target monoaminergic systems.

Animal models are indispensable tools for unraveling the neurobiological mechanisms that underlie anxiety and assessing the behavioral impact of new drug candidates that affect different aspects of anxiety [23–25]. In the present study, we explored the anxiolytic-like effects of quercitrin using the elevated plus maze (EPM), light/dark box (LDB) test, and marble-burying test. The open-field test (OFT) was used to evaluate spontaneous locomotor activity. We investigated the involvement of GABA_A_ and 5-HT_1A_ receptors in the anxiolytic-like properties of quercitrin. To explore the neuropharmacological mechanism, we assessed the influence of quercitrin on the levels of 5-HT and dopamine and their metabolites. Our results showed that quercitrin exerted an anxiolytic-like effect.

## 2. Materials and Methods

### 2.1. Animals

All of the experiments were performed using male ICR mice (19–21 g), which were obtained from the Vital River Company, Beijing, China. The mice were housed five per cage (25 cm × 15 cm × 14 cm) in a temperature- (23 ± 1°C) and humidity- (55%  ± 5%) controlled room under a 12 h/12 h light/dark cycle (lights on 7:00 AM–7:00 PM) with* ad libitum* access to food and water. All of the experiments were performed between 8 AM and 2 PM. The mice were introduced to the quiet experimental room under dim red light at least 1 h before testing. The experimental procedures were approved by the Animal Care and Use Committee of the Institute of Psychology, Chinese Academy of Sciences, and were in accordance with the National Institutes of Health Guide for Care and Use of Laboratory Animals.

### 2.2. Materials

Quercitrin was purchased from the China Food and Drug Inspection Institute. Diazepam was obtained from Yimin Pharmaceutical Factory (Beijing, China). 5-HT, 5-hydroxy-3-indoleacetic acid (5-HIAA), dopamine, 3,4-dihydroxyphenylacetic acid (DOPAC), and homovanillic acid (HVA) were purchased from Sigma (St. Louis, MO, USA). All of the other reagents were of analytical grade.

### 2.3. Treatments and Groups

**Figure 1:**
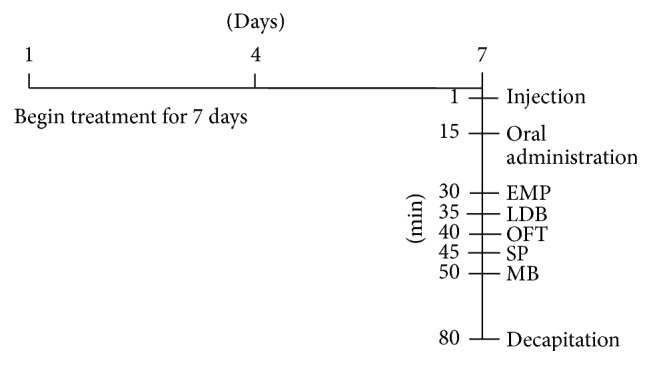
Schematic illustration of the experimental design. The vehicle (control), quercitrin (2.5, 5.0, and 10.0 mg/kg), and diazepam (DZP, 2 mg/kg) were orally administered daily for 7 days. On the last day, the antagonists flumazenil (F; 3 mg/kg, i.p.) and WAY-100635 (W; 0.5 mg/kg, i.p.) were injected 15 min before the oral treatments. The behavioral tests (EPM, LDB, OFT, SP, and MB tests) were performed, and tissue samples were collected on the same day.

The mice (*n* = 15 per group) were randomly assigned to eight experimental groups: vehicle control group, diazepam group, quercitrin groups (2.5, 5.0, and 10.0 mg/kg), diazepam + flumazenil group, quercitrin + flumazenil group, and quercitrin + WAY-100635 group. To evaluate the anxiolytic effects of quercitrin, quercitrin (2.5, 5.0, and 10.0 mg/kg) and diazepam (2.0 mg/kg) were orally administered to the mice for 7 days. To explore the involvement of GABA_A_ and 5-HT_1A_ receptors in the effects of quercitrin, quercitrin (5.0 mg/kg) or diazepam (2.0 mg/kg) was administered to the mice for 7 days. On the last day, the GABA_A_ receptor antagonist flumazenil (Sigma, St. Louis, MO, USA; 0.5 mg/kg, i.p.) or 5-HT_1A_ receptor antagonist WAY-100635 (Sigma, St. Louis, MO, USA; 3 mg/kg, i.p.) was coadministered with quercitrin, and flumazenil was coadministered with diazepam 15 min before oral administration. The behavioral testing was conducted 30 min after oral administration. Diazepam at a dose of 2 mg/kg was chosen as a positive control drug. The dose and route of administration for diazepam and quercitrin were based on previous studies [8, 9, 26–28]. Quercitrin and diazepam were suspended in 0.9% physiological saline solution that contained 1% Tween 80, and flumazenil and WAY-100635 were dissolved in physiological saline. Control animals received vehicle orally and by injection. All of the drugs were prepared immediately before use and administered orally in a volume of 0.5 mL/25 g body weight for 7 days. All of the behavioral tests were performed on the 7th day of treatment. All groups of animals underwent all behavior tests. The study design is shown in Figure 1.

### 2.4. Experiments

#### 2.4.1. Elevated Plus Maze

This validated test has been widely used to measure anxiety in rodents [24]. The EPM consisted of two open arms (30 cm × 5 cm × 15 cm) and two closed arms (30 cm × 5 cm) that were connected by a central platform (5 cm × 5 cm). It was elevated 45 cm above the floor. The open arms had a low edge (0.25 cm) that provided additional grip for the animals. The test was performed 30 min after oral administration. The mice were individually placed in the center of the maze, facing an open arm. The number of entries into the open and closed arms and time spent on the open and closed arms were recorded by AVTAS 3.0 software (Anilab, Ningbo, China) during a 5 min observation period. The percentage of entries into the open arms (% OE = entries into open arms/entries into open and closed arms) and percentage of time spent on the open arms (% OT = time spent on the open arms/time spent on the open and closed arms) were treated as an index of anxiety. Animals were excluded if they fell from the maze. The maze was cleaned thoroughly between trials using 10% ethanol.

#### 2.4.2. Light/Dark Box Test

The LDB test was performed immediately after the EPM test. When the EPM test was completed, the mouse was immediately placed in the LDB. The apparatus consisted of two compartments, with one-third painted white and two-thirds painted black, which were divided by a plate with a small hole opening at floor level that allowed the mice to pass from one compartment to the other. A 60 W white light was placed 40 cm above the light chamber. The mouse was placed in the light compartment and allowed to freely explore the apparatus for 5 min. The number of transfers from the dark compartment to the light compartment and the time spent in the light compartment were recorded over 5 min by an observer using a chronometer. The observer remained quiet during the entire experiment. The apparatus was thoroughly cleaned between each test.

#### 2.4.3. Open-Field Test

When the LDB test was completed, the mouse was immediately placed in the open field. The open field consisted of a square arena (60 cm × 60 cm). The entire apparatus was enclosed by 25 cm high walls made of black Plexiglas. The arena was illuminated by two 60 W red lamps that were placed over the center of the apparatus. The lamps were close to each other, 120 cm above the floor, and provided 100 lux illumination in the testing room. The test began by placing a single mouse in the middle of the arena and allowing it to move freely for 5 min. The total distance travelled was recorded by an automatic video tracking system and AVTAS 3.0 software (Anilab, Ningbo, China). After each trial, the apparatus was wiped clean with a 10% ethanol solution.

#### 2.4.4. Spontaneous Activity

The spontaneous activity test was performed immediately after the open-field test. The locomotor test apparatus consisted of four clear acrylate test boxes (40 cm width × 40 cm length × 35 cm height) and four standard sound-attenuating cubicles with a camera and infrared lights. The mice were tested in a dim room that was illuminated with a 60 W red light (20 lux). To rule out any possible nonspecific locomotor effects of quercitrin on our measures of anxiety, spontaneous locomotor activity was recorded using a computer-based system (Anilab, Ningbo, China). During the observation period, the software detected infrared light beam breaks. The apparatus was thoroughly cleaned between each test.

#### 2.4.5. Marble-Burying Test

The marble-burying test was performed immediately after the spontaneous activity test. Marble-burying behavior was assessed based on previously published methods [29]. The test was performed using transparent cages (37 cm length × 21 cm width × 15 cm height) that had 5 cm of bedding material and 20 glass marbles (1.5 cm diameter) that were equidistantly distributed throughout the cage. The marbles were placed in a 4 × 5 grid, with no marble closer than 1 cm from the wall of the cage. Testing was performed under normal ambient room lighting (>350 lux). The mice were individually placed in the cage, and transparent plastic was placed on top of the cage to prevent escape. At the end of the 30 min test, the mice were carefully removed from the chamber, and the number of marbles that were buried (i.e., more than two-thirds of the marble was covered by bedding) was determined.

#### 2.4.6. Determination of Monoamines and Metabolites

The mice were decapitated by cervical dislocation immediately after the marble-burying test. The brains were dissected and immediately placed on ice. The tissue samples were weighed and stored at −80°C until homogenization. The brain tissue was manually homogenized with three volumes (w/v) of ice-cold 0.1 M perchloric acid (100 *μ*L/mg wet weight) that contained 0.1 mM ethylenediaminetetraacetic acid (EDTA). After homogenization and centrifugation at 12,000 ×g at 4°C for 10 min, 20 *μ*L of the tissue homogenate supernatant was injected directly into a high-performance liquid chromatography (HPLC) system that was equipped with an electrochemical detector (Waters ECD 2465, Milford, Massachusetts, USA). The mixed standard was used as a reference. The levels of monoamines (5-HT, dopamine, 5-HIAA, DOPAC, and HVA) in the samples were expressed as nanograms per gram fresh weight of tissue [30]. The HPLC system included a reversed-phase C18 column (2.1 mm × 150 mm, 3 *μ*m, Waters Atlantis). The mobile phase consisted of 50 mM citric acid-sodium citrate (pH 3.5), 0.3 mM Na_2_-EDTA, 1.8 mM dibutylamine, and 4% methanol. The flow rate was 0.35 mL/min, and the detector potential was +0.75 V.

### 2.5. Statistical Analysis

The data are expressed as mean ± SEM. The statistical analysis was performed using one- or two-way analysis of variance (ANOVA) followed by the Student-Newman-Keuls post hoc test and GraphPad Prism 5.0 software. In cases of significant variation, the individual values were compared using Dunnett's test. Values of *p* < 0.05 were considered statistically significant.

## 3. Results

### 3.1. Effect of Quercitrin in the Elevated Plus Maze

**Figure 2:**
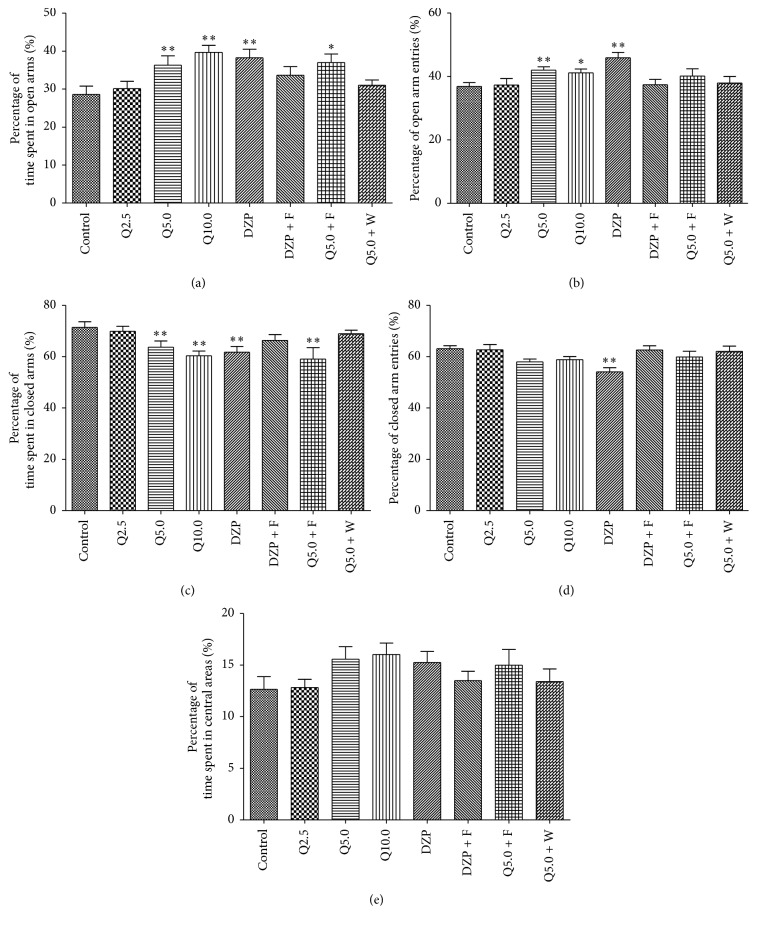
Behavioral performance of mice in a 5 min session in the elevated plus maze performed 0.5 h after the injection of vehicle (control, p.o.), quercitrin (2.5, 5.0, and 10.0 mg/kg, p.o.), diazepam (DZP, 2 mg/kg, p.o.), flumazenil (F; 3 mg/kg, i.p., 15 min after p.o. administration of positive control or quercitrin, Q), and WAY-100635 (W; 0.5 mg/kg, i.p., 15 min before p.o. administration of quercitrin, Q). (a) Percentage of time spent in the open arms, (b) percentage of open arm entries, (c) percentage of time spent in the closed arms, (d) percentage of closed arm entries, and (e) percentage of time spent in central areas. Columns represent the means ± SEM; *n* = 15 mice. ^⁎^ *p* < 0.05 and ^⁎⁎^ *p* < 0.01 compared to the control group.

The one-way ANOVA revealed significant differences between groups in the time spent on the open arms (*F*_7,127_ = 4.330, *p* < 0.01, Figure 2(a)), percentage of open arm entries (*F*_7,127_ = 3.123, *p* < 0.05, Figure 2(b)), percentage of the time spent in the closed arms (*F*_7,127_ = 4.140, *p* < 0.01, Figure 2(c)), percentage of closed arm entries (*F*_7,127_ = 3.342, *p* < 0.01, Figure 2(d)), and percentage of the time spent in the central areas (*F*_7,127_ = 1.692, *p* > 0.05, Figure 2(e)). As shown in Figure 2(a), treatment with 5.0 and 10.0 mg/kg quercitrin significantly increased the percentage of time spent on the open arms (both *p* < 0.01). Diazepam (2 mg/kg) significantly increased the percentage of time spent on the open arms compared with the control group (*p* < 0.01). As shown in Figure 2(b), diazepam (both *p* < 0.01) and 5.0 and 10.0 mg/kg quercitrin (*p* < 0.01 and *p* < 0.05, resp.) significantly increased the number of entries into the open arms compared with the control group. As shown in Figure 2(c), treatment with 5.0 and 10.0 mg/kg quercitrin significantly decreased the percentage of time spent on the closed arms (both *p* < 0.01) as well as diazepam (*p* < 0.01). As shown in Figure 2(d), diazepam (both *p* < 0.01) significantly decreased the number of entries into the open arms compared with the control group whereas the entries into the closed arms in the quercitrin-treated group did not produce significant differences compared to the control group. No significant differences were found in the percentage of time spent in central areas (*p* > 0.05, Figure 2(e)).

### 3.2. Effect of Quercitrin in the Light/Dark Box Test

**Figure 3:**
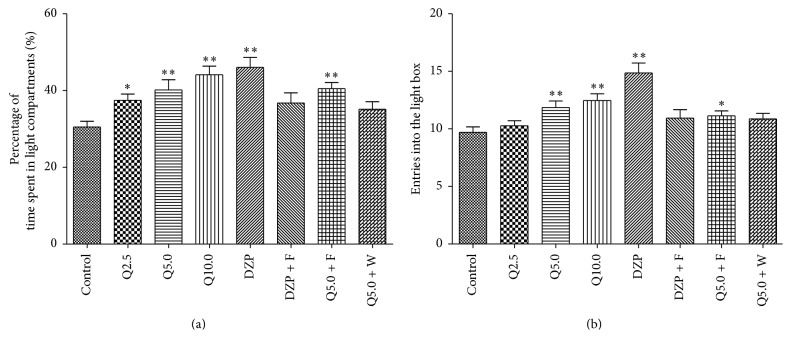
Behavioral performance of mice in a 5 min session in the light and dark box performed 0.5 h after the injection of vehicle (control, p.o.), quercitrin (2.5, 5.0, and 10.0 mg/kg, p.o.), diazepam (DZP, 2 mg/kg, p.o.), flumazenil (F; 3 mg/kg, i.p., 15 min after p.o. administration of positive control or quercitrin, Q), and WAY-100635 (W; 0.5 mg/kg, i.p., 15 min before p.o. administration of quercitrin, Q). (a) Percentage of time spent in light compartment and (b) entries into the light box. Columns represent the means ± SEM; *n* = 15 mice. ^⁎^ *p* < 0.05 and ^⁎⁎^ *p* < 0.01 compared to the control group.

The one-way ANOVA revealed significant differences between groups in the time spent in the light compartment (*F*_7,127_ = 5.174, *p* < 0.01, Figure 3(a)) and number of entries into the light compartment (*F*_7,127_ = 7.217, *p* < 0.01, Figure 3(b)). As shown in Figure 3(a), 2.5, 5.0, and 10.0 mg/kg quercitrin significantly increased the time spent in the light compartment (all *p* < 0.01). Diazepam (2 mg/kg) significantly increased the percentage of time spent in the light compartment compared with the control group (*p* < 0.01). Additionally, 5.0 and 10.0 mg/kg quercitrin (both *p* < 0.01) and diazepam (both *p* < 0.01) significantly increased the number of entries into the light compartment compared with the control group.

### 3.3. Effect of Quercitrin in the Marble-Burying Test

**Figure 4:**
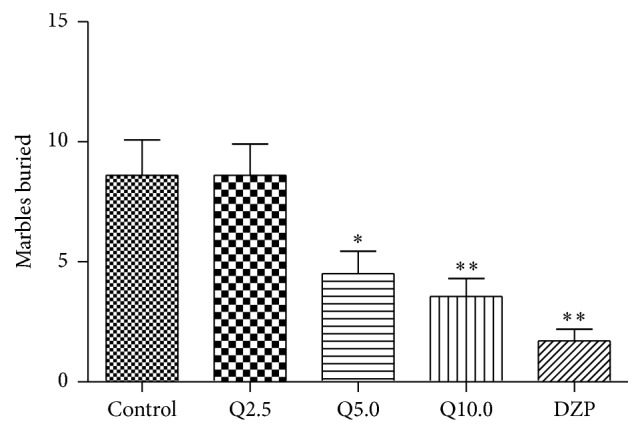
Marbles buried number of mice in a 30 min session in the marble-burying test performed 1.0 h after the injection of vehicle (control, p.o.), quercitrin (2.5, 5.0, and 10.0 mg/kg, p.o.), and diazepam (DZP, 2 mg/kg, p.o.). Columns represent the means ± SEM; *n* = 15 mice. ^⁎^ *p* < 0.05 and ^⁎⁎^ *p* < 0.01 compared to the control group.

The one-way ANOVA revealed significant differences between groups in the number of marbles buried (*F*_4,79_ = 8.476, *p* < 0.01, Figure 4). Diazepam (2.0 mg/kg) and quercitrin (5.0 and 10.0 mg/kg) significantly decreased the number of marbles buried compared with the control group (*p* < 0.01), whereas 2.5 mg/kg quercitrin had no effect.

### 3.4. Effect of Quercitrin in the Open-Field Test

**Figure 5:**
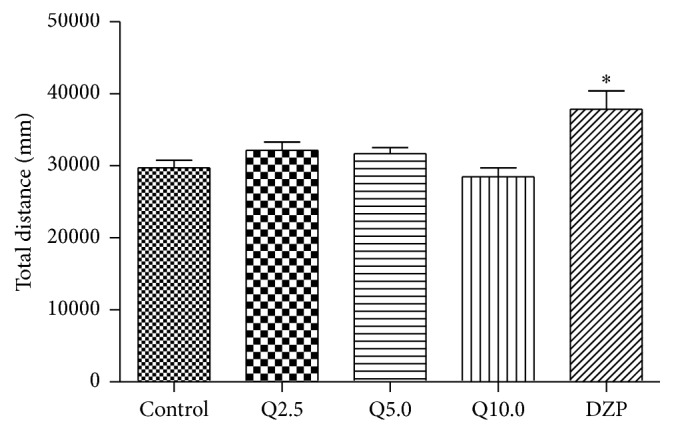
Total moving distance of mice in a 5 min session in the open-field test performed 0.5 h after the injection of vehicle (control, p.o.), quercitrin (2.5, 5.0, and 10.0 mg/kg, p.o.), and diazepam (DZP, 2 mg/kg, p.o.). Columns represent the means ± SEM; *n* = 15 mice. ^⁎^ *p* < 0.05 and ^⁎⁎^ *p* < 0.01 compared to the control group.

The one-way ANOVA revealed significant differences between groups in the total distance travelled (*F*_4,79_ = 5.813, *p* < 0.01, Figure 5). As shown in Figure 5, diazepam (2.0 mg/kg) significantly increased the total distance travelled compared with the control group (*p* < 0.01), whereas no differences were found between the quercitrin-treated groups and control group.

### 3.5. Effect of Quercitrin in the Spontaneous Locomotor Activity Test

**Figure 6:**
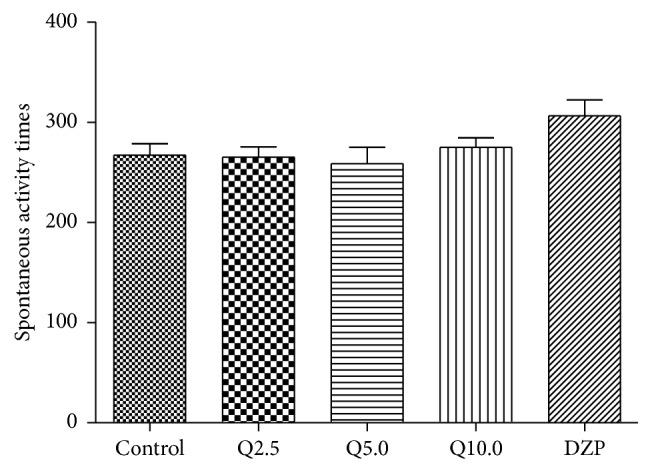
Number of activity times of mice in a 5 min session in the spontaneous activity test performed 0.5 h after the injection of vehicle (control, p.o.), quercitrin (2.5, 5.0, and 10.0 mg/kg, p.o.), and diazepam (DZP, 2 mg/kg, p.o.). Columns represent the means ± SEM; *n* = 15 mice. ^⁎^ *p* < 0.05 and ^⁎⁎^ *p* < 0.01 compared to the control group.

The one-way ANOVA revealed significant differences between groups in spontaneous locomotor activity (*F*_4,79_ = 2.075, *p* < 0.05, Figure 6). Diazepam (2 mg/kg) significantly increased spontaneous locomotor activity compared with the control group (*p* < 0.01). No differences in spontaneous locomotor activity were found between the quercitrin-treated groups and control group (Figure 6).

### 3.6. Influence of WAY-100635 on the Anxiolytic-Like Effects of Quercitrin

To determine whether the anxiolytic-like effects of quercitrin involve the serotonergic system, especially 5-HT_1A_ receptors, diazepam- (2.0 mg/kg) and quercitrin- (5.0 mg/kg) treated mice were pretreated with the 5-HT_1A_ receptor antagonist WAY-100635. The two-way ANOVA revealed significant differences between different treatments in the percentage of the time spent in the open arm (*F*_4,89_ = 3.914, *p* < 0.05, Figure 2(a)) and in the open arms entries (*F*_4,89_ = 5.564, *p* < 0.01, Figure 2(b)) and percentage of the time spent in the closed arm (*F*_4,89_ = 3.914, *p* < 0.05, Figure 2(c)) and in the closed arms entries (*F*_4,89_ = 5.565, *p* < 0.01, Figure 2(d)). The two-way ANOVA revealed significant differences between different pretreatments in the open arms entries (*F*_4,89_ = 5.520, *p* < 0.01, Figure 2(b)) and in the closed arms entries (*F*_4,89_ = 5.510, *p* < 0.01, Figure 2(d)), while there were no significant differences between different pretreatments in the percentage of the time spent in the open arm (*F*_4,89_ = 2.916, *p* > 0.05, Figure 2(a)) and in the percentage of the time spent in the closed arm (*F*_4,89_ = 2.916, *p* > 0.05, Figure 2(c)). As shown in Figure 2, 5.0 mg/kg quercitrin and diazepam significantly increased the percentage of time spent on the open arms and percentage of entries into the open arms compared with the vehicle-treated control group. 5.0 mg/kg quercitrin and diazepam significantly decreased the percentage of time spent on the closed arms compared to the vehicle-treated control group, but no significant difference was found in the percentage of closed arm entries. Pretreatment with WAY-100635 15 min before drug administration significantly blocked the effects of quercitrin, which significantly decreased the number of entries into the open arms and time spent on the open arms and increased the time spent in the closed arm. In the LDB test, the two-way ANOVA revealed significant differences between different treatments in the percentage of the time spent in the light compartment (*F*_4,89_ = 9.905, *p* < 0.01, Figure 3(a)) and in the light compartment entries (*F*_4,89_ = 12.933, *p* < 0.01, Figure 3(b)), and there were significant differences between different pretreatments in the percentage of the time spent in the light compartment (*F*_4,89_ = 3.605, *p* < 0.05, Figure 3(a)) and in the light compartment entries (*F*_4,89_ = 7.089, *p* < 0.01, Figure 3(b)). 5.0 mg/kg quercitrin and 2.0 mg/kg diazepam significantly increased the time spent in the light compartment and entries into the light compartment (Figure 3). Pretreatment with WAY-100635 15 min before administration significantly blocked the effects of quercitrin, which decreased the time spent in the light compartment and entries into the light compartment. These results indicate that the anxiolytic-like effects of quercitrin were blocked by WAY-100635.

### 3.7. Influence of Flumazenil on the Anxiolytic-Like Effects of Quercitrin

To determine whether the anxiolytic-like effects of quercitrin involve the GABA-ergic system, particularly the benzodiazepine site of GABA_A_ receptors, diazepam- (2.0 mg/kg) and quercitrin- (5.0 mg/kg) treated mice were pretreated with the GABA_A_ receptor antagonist flumazenil. As shown in Figure 2, in the EPM, diazepam and quercitrin (5 mg/kg) significantly increased the percentage of time spent on the open arms and percentage of open arm entries compared with the vehicle-treated control group. Diazepam and quercitrin (5 mg/kg) significantly decreased the percentage of time spent on the closed arms compared to the vehicle-treated control group, but no significant differences were found in the percentage of closed arm entries. Pretreatment with flumazenil before drug administration blocked the effects of diazepam. Pretreatment with flumazenil did not block the effects of quercitrin. In the LDB test (Figure 3), 5.0 mg/kg quercitrin and 2.0 mg/kg diazepam significantly increased the time spent in the light compartment and entries into the light compartment. Pretreatment with flumazenil 15 min before drug administration significantly blocked the effects of diazepam but not quercitrin.

### 3.8. Effects of Quercitrin on Monoamine Neurotransmitters and Their Metabolites

**Figure 7:**
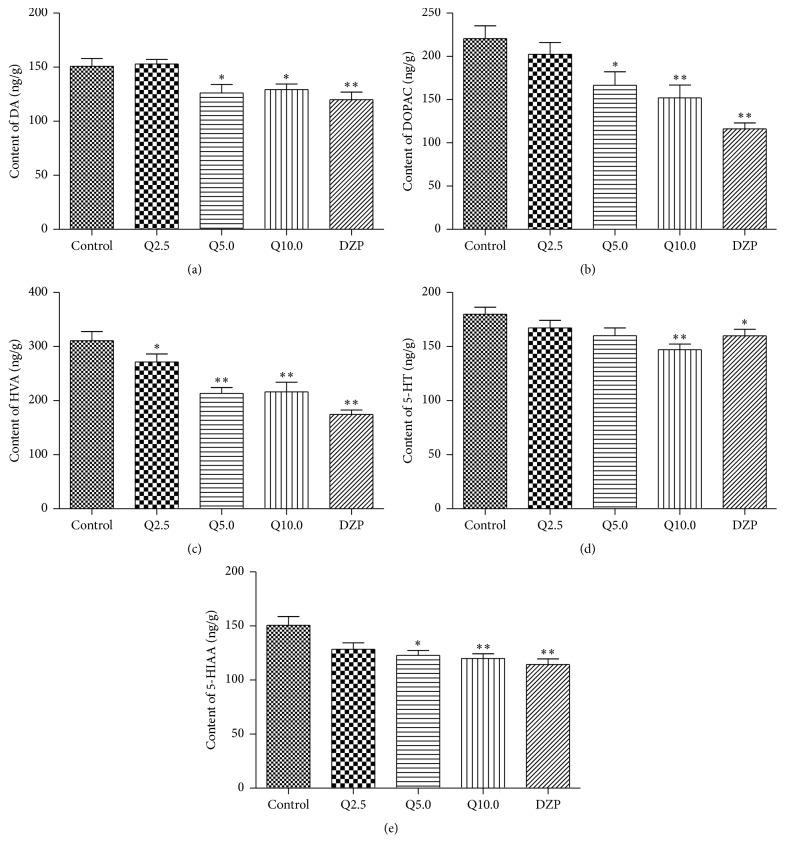
Effects of quercitrin on the monoamines neurotransmitters and their metabolites in the brains of mice. (a) The content of DA, (b) the content of DOPAC, (c) the content of HVA, (d) the content of 5-HT, and (e) the content of 5-HIAA. Results are expressed as mean ± SEM; *n* = 8 mice. ^⁎^ *p* < 0.05 and ^⁎⁎^ *p* < 0.01 compared to the control group.

The one-way ANOVA revealed significant differences in the levels of monoamine neurotransmitters and their metabolites (*F*_4,79_ = 5.362, *p* < 0.01, Figure 7(a);* F*_4,79_ = 9.237, *p* < 0.01, Figure 7(b);* F*_4,79_ = 14.13, *p* < 0.01, Figure 7(c);* F*_4,79_ = 3.507, *p* < 0.01, Figure 7(d);* F*_4,79_ = 5.718, *p* < 0.01, Figure 7(e)). Quercitrin (5.0 mg/kg) significantly decreased dopamine, DOPAC, HVA, and 5-HIAA levels (Figures 7(a)–7(c) and 7(e)); diazepam and quercitrin (10.0 mg/kg) significantly decreased the levels of both monoamines and their metabolites (*p* < 0.01, Figure 7).

## 4. Discussion

Flavonoids are secondary plant metabolites [31] that have a wide range of biological activities, such as antioxidant and anti-inflammatory effects, and readily cross the blood-brain barrier [32]. The anxiolytic effects of many flavone compounds that are derived from plants and synthetic sources, such as luteolin and apigenin, have been reported [33]. Our previous study showed that total flavones contained in* Albizia julibrissin* exerted anxiolytic-like effects [8]. Quercitrin is the main compound of total flavones [10]. The present results suggest that quercitrin exerts anxiolytic-like effects in animal models of anxiety and on the levels of monoamine neurotransmitters and their metabolites. In addition, the effects appeared to be mediated by 5-HT_1A_ receptors.

Anxiety and fear can be induced by the novelty of a situation. They can be evaluated in mice by determining the intensity of behavior in an unfamiliar area, the quantity of unfamiliar food consumption [34], and social interactions with unfamiliar animals. The EPM is based on rodents' natural aversion to height and open spaces [24, 25]. The EPM is considered an ethologically valid animal model of anxiety because it uses natural stimuli (e.g., fear of novel open spaces and fear of balancing on a relatively narrow, raised platform) that can induce anxiety in humans [35]. In the present study, oral quercitrin administration induced an anxiolytic-like effect in mice. Quercitrin increased the percentage of entries into and time spent on the open arms of the EPM, while it decreased the percentage of time spent on the closed arms. These effects were blocked by the 5-HT_1A_ receptor antagonist WAY-100635 and were unaffected by the GABA_A_ receptor antagonist flumazenil. Our results suggest that quercitrin has anxiolytic properties in the EPM, and the 5-HT system may be involved in its effects.

The LDB test is based on rodents' innate aversion to brightly illuminated areas and spontaneous exploratory behavior in response to mild stressors (i.e., a novel environment and light). The effects of classic anxiolytics (e.g., benzodiazepines) and newer anxiolytic-like agents (e.g., serotonergic drugs or drugs that act on neuropeptide receptors) can be detected with this paradigm. In the present study, quercitrin significantly increased the number of transitions between the light and dark compartments and time spent in the light compartment in the LDB test. These results indicate that quercitrin has anxiolytic-like activity. Moreover, the anxiolytic effects were blocked by the 5-HT_1A_ receptor antagonist WAY-100635.

The marble-burying test has been used to screen anxiolytics, including those for obsessive-compulsive disorder. Diazepam has been shown to decrease the number of marbles buried [36]. We found that 5 mg/kg quercitrin significantly decreased the number of marbles buried, indicating that quercitrin has anxiolytic-like activity in the marble-burying test.

To explore the possible mechanism of action of quercitrin, we performed antagonistic experiments. Many anxiolytic drugs exert their behavioral effects by binding to the benzodiazepine site on GABA receptors. In the EPM, we found that the anxiolytic effects of diazepam were completely blocked by flumazenil, whereas flumazenil pretreatment did not influence the effects of 5.0 mg/kg quercitrin. The quercitrin-treated group exhibited anxiolytic-like behavior in the LDB, and these anxiolytic-like effects were not blocked by flumazenil, in which the number of entries into and time spent in the light compartment increased compared with the control group. These results suggest that the anxiolytic-like effects of quercitrin might not be related to GABA_A_ receptors.

5-HT_1A_ receptor agonists, such as buspirone and gepirone, were developed for the treatment of anxiety and depression [37, 38]. To date, the only selective high-affinity antagonist of this receptor is WAY-100635 [39, 40]. In the present study, we confirmed the anxiolytic-like activity of diazepam as previously reported [41] in the EPM. Quercitrin (5.0 mg/kg) produced potent anxiolytic-like effects, and these effects of quercitrin were completely blocked by WAY-100635. Pretreatment with WAY-100635 resulted in percentages of open arm time, percentages of open arm entries, and percentages of closed arm time in the EPM that were not different from the control group. In the LDB test, quercitrin exerted an anxiolytic-like effect. Similarly, the anxiolytic-like effects were blocked by WAY-100635, in which the number of entries into and time spent in the light compartment were not different from the control group. These results suggest that the anxiolytic-like effects of quercitrin might be mediated by 5-HT_1A_ receptors.

The central dopaminergic system is considered the crucial factor in anxiety disorders. Foot-shock and anxiogenic drugs markedly increase cortical dopamine output in normal rats, and chronic treatment with imipramine completely inhibits these changes [42]. The present results are consistent with these reports. Quercitrin significantly reduced the tissue concentration of dopamine in brain homogenates. The levels of DOPAC and HVA, the major metabolites of dopamine, significantly decreased. Therefore, we can speculate that the mechanism of action of quercitrin involves suppression of the synthesis and release of dopamine.

Changes in the serotonergic system were also observed in the present study. According to the classic serotonin hypothesis, anxiety is usually associated with increases in endogenous 5-HT, and anxiolytics tend to decrease endogenous 5-HT [43]. The selective 5-HT_1A_ receptor agonist buspirone exerts anxiolytic effects by decreasing the concentration of 5-HT [44]. The present study is consistent with these reports. Quercitrin significantly decreased 5-HT levels in brain homogenates. The reduction of 5-HT levels might be attributable to the suppression of synthesis or release or an increase in metabolism [45]. Quercitrin also decreased the levels of the primary metabolite of 5-HT, 5-HIAA. These results suggest the quercitrin does not affect the metabolic pathway of 5-HT but decreases its synthesis or release. This mechanism will need to be explored further.

We chose only diazepam as a positive control to evaluate the effects of quercitrin. The 5-HT partial agonist buspirone was not used because it requires 1–4 weeks to show efficacy [46, 47]. Future studies should include buspirone as a positive control and compare its effects with a 5-HT_1A_ receptor agonist. The present results cannot exclude the possibility that other binding sites of GABA and other neurotransmitter systems may play a role in the anxiolytic-like effects of quercitrin.

To reduce the number of animals used, we did not include groups in which WAY-100635 and flumazenil were administered alone. Antagonists usually do not induce any significant changes in behaviors in control animals [48]. In the present study, these drugs were administered by the same routes as quercitrin. We did not assess the effects of other routes of administration on anxiety-related behavior, nor did we assess bioavailability. These remain to be studied in the future.

## 5. Conclusions

In summary, the present results indicate that quercitrin exerts anxiolytic-like effects without affecting locomotor activity in mice. The anxiolytic effects of quercitrin appear to be mediated by 5-HT_1A_ receptors and not involve the benzodiazepine binding site of GABA receptors. Our neurochemical studies suggest that these effects are mediated through the modulation of monoamine neurotransmitter levels. The present results suggest the potential usefulness of quercitrin for the treatment of anxiety disorders.
